# Identification and Molecular Characterization of Shamonda Virus in an Aborted Goat Fetus in South Africa

**DOI:** 10.3390/pathogens12091100

**Published:** 2023-08-28

**Authors:** Miné van der Walt, Matshepo E. Rakaki, Caitlin MacIntyre, Adriano Mendes, Sandra Junglen, Cherise Theron, Tasneem Anthony, Nicolize O’Dell, Marietjie Venter

**Affiliations:** 1 Zoonotic Arbo- and Respiratory Virus Research Program, Centre for Viral Zoonoses, Department of Medical Virology, University of Pretoria, Pretoria 0084, South Africa; mvdwalt89@gmail.com (M.v.d.W.); rakakime@gmail.com (M.E.R.); cdm.macintyre@gmail.com (C.M.); adriano.mendes288@gmail.com (A.M.); 2 Institute of Virology, Charité Universitätsmedizin Berlin, Corporate Member of Freie Universität Berlin, Humboldt-Universität zu Berlin, and Berlin Institute of Health, Charitéplatz 1, 10117 Berlin, Germany; sandra.junglen@charite.de; 3 Western Cape Provincial Veterinary Laboratory, Stellenbosch 7600, South Africa; cherise.swanepoel17@gmail.com (C.T.); tasneem.anthony@westerncape.gov.za (T.A.); 4 Department of Paraclinical Science, Section Pathology, Faculty of Veterinary Science, University of Pretoria, Onderstepoort, Pretoria 0002, South Africa; nicolize.odell@up.ac.za

**Keywords:** Shuni virus, Shamonda virus, neurological, abortion, reassortment

## Abstract

Viruses in the *Orthobunyavirus* genus, *Peribunyaviridae* family, are associated with encephalitis, birth defects and fatalities in animals, and some are zoonotic. Molecular diagnostic investigations of animals with neurological signs previously identified Shuni virus (SHUV) as the most significant orthobunyavirus in South Africa (SA). To determine if other orthobunyaviruses occur in SA, we screened clinical specimens from animals with neurological signs, abortions, and acute deaths from across SA in 2021 using a small (S) segment Simbu serogroup specific TaqMan real-time reverse transcription polymerase chain reaction (RT-PCR). Positive cases were subjected to Sanger sequencing and phylogenetic analysis to identify specific viruses involved, followed by next-generation sequencing (NGS) and additional PCR assays targeting the medium (M) segment and the large (L) segment. In total, 3/172 (1.7%) animals were PCR positive for Simbu serogroup viruses, including two horses with neurological signs and one aborted goat fetus in 2021. Phylogenetic analyses confirmed that the two horses were infected with SHUV strains with nucleotide pairwise (p-) distances of 98.1% and 97.6% to previously identified strains, while the aborted goat fetus was infected with a virus closely related to Shamonda virus (SHAV) with nucleotide p-distances between 94.7% and 91.8%. Virus isolation was unsuccessful, likely due to low levels of infectious particles. However, phylogenetic analyses of a larger fragment of the S segment obtained through NGS and partial sequences of the M and L segments obtained through RT-PCR and Sanger sequencing confirmed that the virus is likely SHAV with nucleotide p-distances between 96.6% and 97.8%. This is the first detection of SHAV in an aborted animal in SA and suggests that SHAV should be considered in differential diagnosis for abortion in animals in Southern Africa.

## 1. Introduction

The *Bunyavirales* order represents a vast and diverse group of ribonucleic acid (RNA) viruses, encompassing 12 families, 46 genera, and over 350 species [1]. Among these families, the *Orthobunyavirus* genus, belonging to the *Peribunyaviridae* family, comprises approximately 170 species and is the largest and most diverse family in this order [1]. These viruses are primarily arthropod-borne (arbo-) viruses transmitted by ticks, sand flies, and mosquitoes that feed on vertebrate hosts [2,3]. The *Orthobunyavirus* genus currently consists of approximately 18 serogroups based on complement-fixation studies. However, viruses in the same serogroup may exhibit different evolutionary patterns [4]. In the context of Simbu serogroup viruses, phylogenetic research has revealed their classification into two distinct clades, namely clade A and clade B [4]. Oropouche virus (OROV) and Manzanilla virus are among the viruses in clade A, whereas the pathogens in clade B include Akabane virus, Shuni virus (SHUV), Shamonda virus (SHAV), Schmallenberg virus (SBV), and Sathuperi virus (SATV) [4]. Understanding the placement of SHAV within this serogroup is essential for elucidating its evolutionary relationships and pathogenic potential. Three distinct linear, negative sense, single-stranded RNA segments make up the genome of orthobunyaviruses, namely the small (S) segment encoding for the nucleocapsid (N) protein, the medium (M) segment encoding for the envelope glycoprotein, which consists of two glycoproteins, and the large (L) segment encoding for the RNA-dependent RNA polymerase (RdRp), respectively [5,6,7,8]. The M segment is the most variable segment and is a common substrate for reassortment between orthobunyaviruses, while the S segment is highly conserved [9,10].

Notably, the Simbu serogroup is of significant interest as it contains zoonotic pathogens capable of infecting humans and animals [11,12]. Many of these viruses induce subclinical infections, whereas others cross the placenta and cause fetal infections that result in abortion, stillbirth, premature birth, and congenital defects in offspring [13,14]. This pathology is more frequently detected in animals. Moreover, certain Simbu serogroup viruses have shown neuroinvasiveness in humans [15]. Among the viruses in the Simbu serogroup, SBV is an example of a more recently emerging orthobunyavirus. First detected in 2011, SBV has since expanded widely throughout Europe, causing death in newborn sheep, goats, and calves [16,17]. Serological studies have also suggested the presence of antibodies against SBV in cattle in the Otjozondjupa region of Namibia in South Africa (SA) [18]. In this study in SA, SHUV, another virus from the Simbu serogroup, has been detected in horses, wildlife, and humans with neurological infections in SA [15,19,20]. Notably, SHUV also recently emerged in Israel, where it has been associated with neurological infections and birth defects in livestock [21,22]. Oropouche fever, a febrile illness caused by OROV, is another example of a virus in the Simbu serogroup that has caused numerous outbreaks of febrile illness in Central and South America, infecting over half a million individuals over the past four decades [11].

In this broader investigation into Simbu serogroup viruses, a specific focus of significant interest is directed towards SHAV, a member of this serogroup. Originally isolated from a bovine blood sample in Nigeria in 1965, SHAV was later detected in *Culicoides* biting midges in Japan 37 years later and in the Gauteng province of SA between 2012 and 2017 [23,24,25,26]. SHAV is one of the Simbu serogroup viruses that seem to replicate within the fetus by crossing the ruminant placenta and, therefore, can potentially induce miscarriage and congenital abnormalities in the progeny [26]. Although no evidence currently associates SHAV with human diseases, understanding its characteristics is crucial, given the risk of zoonotic transmission.

Through syndromic surveillance in animals with neurological signs or unexplained abortions, this study focused on detecting emerging or reemerging orthobunyaviruses in the Simbu serogroup in SA. Here, we report the first detection of SHAV in animals in SA and aim to molecularly characterize the SHAV strain by generating full or partial genome sequencing data for phylogenetic analyses. These findings provide insights into the potential emergence, spread and evolution of this virus in Southern Africa.

## 2. Materials and Methods

### 2.1. Clinical Sampling

Animal clinical specimens across SA were submitted to the Zoonotic Arbo- and Respiratory Virus (ZARV) research program at the Center for Viral Zoonoses, Department of Medical Virology, University of Pretoria from January to December 2021. These submissions were made as part of a collaborative arbovirus surveillance program involving veterinarians, virologists, and veterinary pathology laboratories. Ethylenediamine tetra-acetic acid (EDTA) blood, plasma, serum, cerebrospinal fluid (CSF), and various organs, including brain, lung, spleen, and liver specimens, were submitted by veterinarians from animals that fit a case definition of acute febrile disease with or without neurological or respiratory signs, abortion, and acute death. Each case was accompanied by a submission form that included demographic, geographical and clinical data.

### 2.2. Specimen Processing and RNA Extraction

RNA extraction was performed on clinical specimens under biosafety level 3 conditions using the QIAamp^®^ Viral RNA Mini Kit (Qiagen, Valencia, CA, USA). A volume of 140 µL of EDTA blood, plasma, serum, or CSF specimens was extracted according to the manufacturer’s instructions. Tissue specimens were processed into a homogenous mixture (200 µL Lysis Buffer and a 30 g cube of the specimen) by using the Qiagen Tissue lyser II (Qiagen) at a frequency of 30 hertz for three minutes (min). Homogenates were subjected to centrifugation at 13,000× g for five min, after which the RNeasy^®^ Mini Kit (Qiagen) was followed to extract RNA from 200 µL of the supernatant.

### 2.3. PCR Assays Used for the Screening of Clinical Specimens

Multiple reverse transcription polymerase chain reaction (RT-PCR) assays using primers and probes specific to the S, M and L segments were used to obtain partial fragments. These are described below in Table 1. PCR reaction conditions are described in Table S1 in the Supplementary Materials.

### 2.4. Molecular Analysis and Sequencing

Primers designed against SHAV, SBV, SATV, Douglas virus (DOUV), and Thimiri virus (THIV) were utilized for overlapping S, M and L segment multiplex amplicon sequencing. The S segment was amplified in two sections (497 base pairs (bp) and 278 bp), while multiple fragments (~500 bp each) of the M and L segments were targeted. The primers were divided into two pools at a working concentration of 10 µM. RNA extraction was followed by complementary deoxyribonucleic acid (cDNA) synthesis using the SuperScript™ III First-Strand Synthesis System (Thermo Fisher Scientific, Waltham, MA, USA) with 8 µL of RNA.

Amplification PCRs were carried out with the iProof™ High-Fidelity PCR Master Mix kit (Bio-Rad Laboratories, Hercules, CA, USA) using 5 µL of cDNA, 12.5 µL of iProof HF Master Mix, and 5 µL of primer pool 1 or 2 at a final concentration of 0.015 µM per individual primer. Primers used for the S and L segments are shown in Supplementary Table S2. The PCR reaction conditions included an initial denaturation at 98 °C for 3 min, followed by 35 cycles of 98 °C for 10 s, 45 °C for 30 s and, 72 °C for 15 s, 72 °C for 10 min and a 4 °C cooling down step. The resulting PCR products were purified using Agencourt AMPure XP beads (Beckman Coulter, Brea, CA, USA), and their concentration was determined using the Qubit^®^ 3.0 Fluorometer (Life Technologies, Carlsbad, CA, USA). Band verification was conducted on a TapeStation (Agilent, Santa Clara, CA, USA).

Illumina libraries were constructed from these PCR products using the Illumina DNA prep kit (Illumina Inc., San Diego, CA, USA). Subsequently, Illumina sequencing was conducted on an iSeq 100 system (Illumina Inc.).

Additionally, PCR products were observed on agarose gel, followed by purification using the Zymoclean™ Gel DNA Recovery kit (Zymo Research, Tustin, CA, USA). Concentration measurements were performed using the Qubit^®^ 3.0 Fluorometer. Nucleotide sequencing was carried out using the Sanger sequencing method and the BigDye Terminator v3.1 Cycle Sequencing Kit (Thermo Fisher Scientific) on an ABI3500xL Genetic analyzer (Applied Biosystems, Thermo Fisher Scientific) at the DNA Sanger Sequencing facility, Faculty Natural and Agricultural Sciences, University of Pretoria.

### 2.5. Sequence Analysis and Depository

CLC Main Workbench version 8.0.1 was used to edit sequence data (https://www.qiagenbioinformatics.com (accessed on 23 June 2023)). BLAST was used to compare the obtained sequences to the GenBank database (Basic Local Alignment Search Tool, www.ncbi.nlm.nih.gov/blast (accessed on 23 June 2023)), and the online version of MAFFT was used to generate multiple sequence alignments using default settings (http://mafft.cbrc.jp/alignment/server/index.html (accessed on 23 June 2023)). Phylogenetic analyses were performed by keeping parameters for the maximum likelihood as default and supported with 1000 bootstrap replicates using MEGA XI software (Molecular Evolutionary Genetics Analysis, https://www.megasoftware.net/ (accessed on 23 June 2023)).

Partial S, M, and L sequences for SHAV were deposited into the National Center for Biotechnology Information. GenBank accession numbers for these partial fragments are OR249959-OR249961.

## 3. Results

### 3.1. Detection of Orthobunyaviruses in Animals with Neurological and Febrile Signs

From January to December 2021, 172 specimens were submitted and tested for Simbu serogroup viruses. A total of 3/172 (1.7%) clinical specimens from animals fitting the case definition and submitted in 2021 tested positive for orthobunyaviruses in the Simbu serogroup, using the S segment-specific TaqMan real-time RT-PCR assay [20].

Cases 1 and 2 involved brain specimens originating from horses in the Gauteng province, presenting neurological signs such as ataxia and head tilting. While these cases provide valuable insights, it is essential to underscore that they are not the primary focal point of this study.

Significantly, Case 3 emerged within the Western Cape region, involving an aborted goat fetus from one of fifty does that experienced abortion during two weeks (Table 2). Among these abortion cases, specimens from this single case were submitted for analysis, including brain, lung, liver, and spleen. Histopathological examination of the lung specimen by the state pathologist revealed meconium aspiration, indicative of fetal distress, along with widespread mononuclear perivascular cuffing, which was observed throughout the various meninges and brain fragments evaluated, indicating virus-induced meningoencephalitis as indicated in the postmortem report. Notably, the macroscopic examination yielded no significant findings aside from the anticipated complete atelectasis of the lungs of the aborted fetus.

### 3.2. Virus Identification

**Figure 1:**
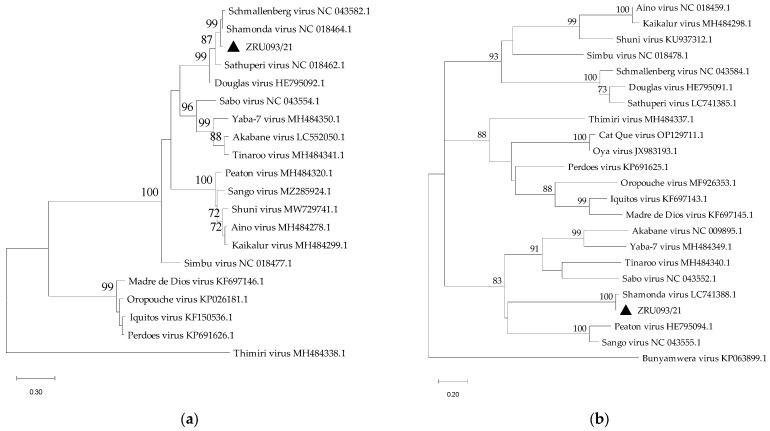

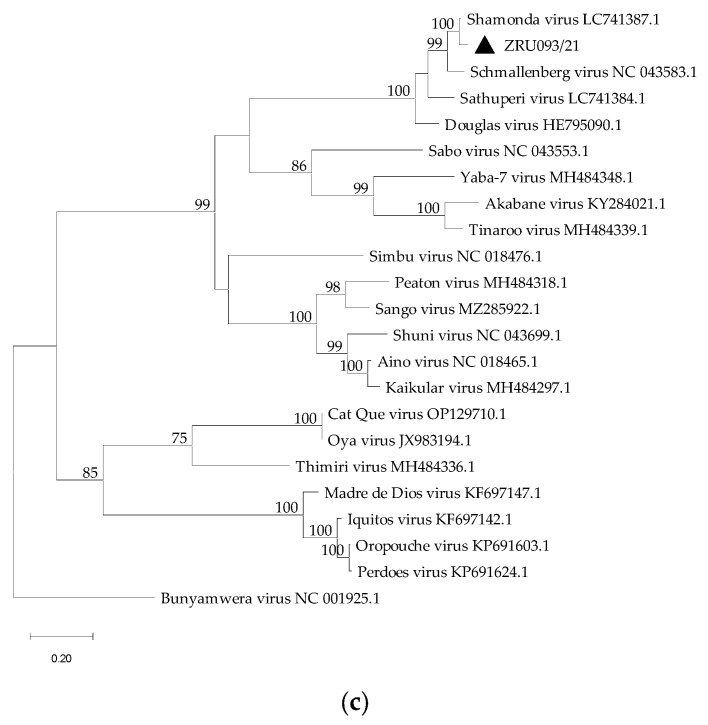
Phylogenetic trees of the nucleotide sequences of partial fragments of the S (**a**), M (**b**) and L (**c**) segments of orthobunyaviruses in the Simbu serogroup. GenBank accession numbers are included, and ZRU093/21 (OR249959.1-OR249961.1) is indicated by a triangle. The percentage of 1000 bootstrap replicates is indicated by the numbers at each node (only values of 70 and higher are shown). Nucleotide substitutions per site are indicated by a scale bar. Thimiri virus was used as an outgroup for the phylogenetic tree in (**a**), and Bunyamwera virus was used as an outgroup for the phylogenetic trees in (**b**,**c**).

Sanger sequencing of the RT-PCR products followed by BLAST and maximum likelihood phylogenetic analysis of orthobunyaviruses in the Simbu serogroup were used for the identification of individual orthobunyaviruses (phylogenetic trees are shown in Figure 1). Nucleotide identities for the S segment of the two horse cases were 96% (98 bp fragment) and 91.3% (97 bp fragment) to SHUV (MK114086.1), respectively, with nucleotide pairwise (p-) distances of 98.1% and 97.6%, respectively. ZRU093/21, which was initially detected at a cycle threshold of 29.68 and 36.25 on the brain and lung specimens, respectively, was further characterized using the S segment Simbu serogroup conventional nested RT-PCR assay, resulting in a larger product of approximately 268 bp. The liver and spleen specimens were negative for orthobunyaviruses from the Simbu serogroup. Initial BLAST analysis showed alignment to SHAV (LC741389.1) and SBV with percentage identities of 87.4% and 85.1%, respectively, while nucleotide p-distances were 94.7% and 91.8%.

### 3.3. Sequence Characterization and Attempted Full Genome Sequencing

Multiple efforts were undertaken to acquire the full genome sequence of the SHAV strain detected in ZRU093/21. Viral isolation was unsuccessful after three passages on Vero and baby hamster kidney (BHK) cell lines. A sequence-independent, single-primer amplification (SISPA) protocol was attempted on the brain tissue, which yielded no orthobunyavirus reads [28]. For this reason, we attempted an amplicon-based approach in which we designed primers against several Simbu serogroup viruses (SHAV, SBV, SATV, DOUV and THIV) to generate overlapping fragments across the three segments of the genome. Due to the limited amount of RNA available from specimen ZRU093/21, it was not possible to test individual primers but pooled primers for each segment. PCR products from amplification PCRs were sequenced on the iSeq 100 system and mapped to SHAV (GenBank accession numbers: LC741389.1 (S segment), LC741388.1 (M segment), LC741387.1 (L segment). Although we were unable to determine the full genome sequence of the virus, partial reads were obtained that confirmed the virus as SHAV. No reads were detected for the M segment. Multiple fragments across the L segment were obtained with multiple attempts, although not long enough or sufficient to differentiate between the Simbu serogroup viruses due to the high similarity of this fragment. However, larger fragments were obtained across the L segment with a maximum of 265 reads and an average coverage of 13.4%.

Next-generation amplicon sequencing also resulted in a region of the S segment (504 bp) with 2247 reads and an average coverage of 63.7%. A consensus sequence was generated by adding S segment sequences obtained via Sanger sequencing to the next-generation sequencing (NGS) fragment to create a final S segment fragment of 671 bp. To generate more sequence data, we turned back to traditional PCR techniques by designing nested PCR assays for the M segment (Table 1) and a previously published PCR assay for the L segment [27]. We obtained partial fragments for the M and L segments of 421 bp (positions 2705–3125) and 457 bp (positions 2974–3432), respectively. A consensus sequence was generated for the L segment, resulting in a 929 bp fragment. Therefore, in total, we identified 2726 bp of the SHAV genome (22.7%), 671 bp (80.0%) of the S segment, 421 bp (9.8%) of the M segment and 1634 bp (23.7%) of the L segment.

### 3.4. Phylogenetic Analysis

After initial BLAST analysis, the closest matched virus sequences and other related sequences were used for phylogenetic and p-distance analysis. Nucleotide (nt) and amino acid (aa) p-distances were determined for the 671 bp fragment of the S segment, the 421 bp fragment of the M segment and the 929 bp fragment of the L segment of ZRU093/21 (Supplementary Materials, Tables S3–S5).

**Figure 2:**
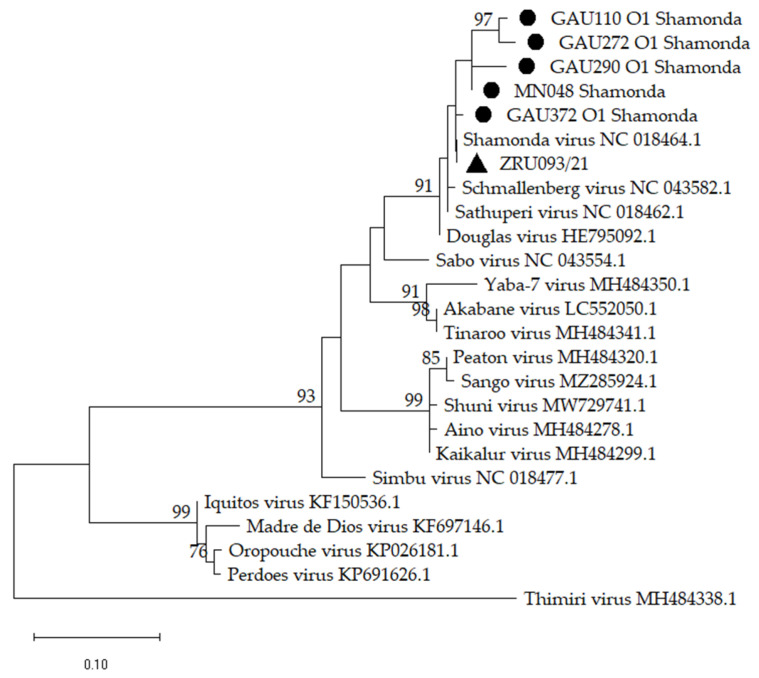
A phylogenetic tree of the Shamonda virus (SHAV) small (S) segment nucleotide sequences obtained from *Culicoides* midges [25] and the aborted goat fetus from this study with the S segment nucleotide sequences of orthobunyaviruses in the Simbu serogroup. GenBank accession numbers are included. The SHAV sequences from the *Culicoides* midges are indicated by circles, and ZRU093/21 (OR249959.1-OR249961.1) is indicated by a triangle. The percentage of 1000 bootstrap replicates is indicated by the numbers at each node (only values of 70 and higher are shown). Nucleotide substitutions per site are indicated by a scale bar. Thimiri virus was used as an outgroup.

Phylogenetic comparison using the SHAV sequences obtained from a vector surveillance study in *Culicoides* midges conducted between 2012 and 2017 in the northern provinces of SA [25], and an aborted goat fetus in the Western Cape province in 2021 was performed to explore their genetic relationship (Figure 2). The nt and aa p-distances for a short fragment on the S segment ranged between 96.6% and 99.3%, and 96.0% and 100.0%, respectively, suggesting high genetic similarity between these SHAV sequences. Trees for the S, M and L segments were inferred separately and included the prototype species of orthobunyaviruses in the Simbu serogroup (Figure 1). The phylogenetic trees agree with the sequence identity results showing that ZRU093/21 is most closely related to SHAV across all three segments and suggests that ZRU093/21 is a SHAV strain. The next closest ancestral virus is SBV, according to the trees, but only when considering segments S and L. The tree for the M segment places SHAV and ZRU093/21 in a group with the closest common ancestor to PEAV and SANV. It is important to note that while these viruses cluster together, their sequence identity is relatively low, as indicated by the p-distance analysis. In this tree, SBV, DOUV and SATV form a more distantly related group to SHAV. The analysis is based on a limited nt region of less than 500 bp within a larger, approximately 4300 bp segment, which restricts our conclusions. We observe potential genetic relationships among SHAV, SBV, DOUV, and SATV, hinting at a common ancestry and suggesting the possibility of reassortment involving SHAV’s M segment with viruses like PEAV or SANV. Goller et al. [29] previously considered renaming SHAV to PEAV or SANV due to genetic similarities. However, such a change could conflict with the substantial S and L segment homologies linked to SBV.

## 4. Discussion

In this study, we describe the identification of SHAV in an aborted goat fetus from the Western Cape province of SA. This follows the identification of SHAV in *Culicoides* midges in the northern parts of SA (Limpopo and Gauteng provinces) between 2012 and 2017 [25]. This is the first identification of SHAV as a potential cause of abortion in Africa since its discovery in Nigeria in 1965 [23]. The veterinary importance of other Simbu serogroup viruses, such as SBV, and the potential for zoonotic transmission in the case of SHUV emphasizes the importance of characterizing the virus further and defining its molecular epidemiology.

We managed to amplify and sequence 22.7% of the genome, including regions of the S, M, and L fragments that allowed us to confirm the strain as SHAV in all genome fragments. Isolating the virus was unsuccessful on Vero and BHK cells in this study, likely due to the low RNA levels reflected by the high Ct values. Attempts to obtain the full genome directly from brain or lung specimens were unsuccessful. SHAV isolation has previously been achieved on BHK21 hamster lung (HmLu-1) cell lines, but isolation from brain and lung specimens may be more challenging [26]. The tissue specimens were stored and transported considerably, possibly affecting viral RNA integrity. Direct RNA sequencing on brain and lung specimens, followed by DNA depletion and SISPA, and full genome amplicon amplification with NGS did not yield results likely due to low viral load or RNA quality. Metagenomic sequencing provided additional reads, although full genome sequencing from tissue specimens remained challenging due to low viral RNA levels and host RNA contamination. Deeper sequencing may result in full genome-level information but remains expensive and not guaranteed to work on tissue specimens. Further optimizing genome enrichment methods or capture probes for orthobunyaviruses may improve the success of full genome sequencing from clinical specimens.

Sanger and next-generation sequencing allowed us to conduct phylogenetic analysis on all three fragments. Although only sequences of the S segment were available from strains previously identified in *Culicoides* midges in the northern parts of SA, these had the highest similarity to the strain identified in the goat fetus in 2021 in SA. When comparing to sequences on GenBank, the strain was the closest related to a SHAV strain previously identified in Japan (unpublished; GenBank accession numbers: LC741389.1 (S segment), LC741388.1 (M segment), LC741387.1 (L segment)) with 93.40%, 97.37% and 95.67% identity for the S, M and L segments, respectively. The similarity between the partial S and L segments of SHAV and SBV, and the low similarity of the M segment to SBV suggests that ZRU093/21, like other SHAVs, might be a reassortant orthobunyavirus with unique properties [30].

Arbovirus infections are becoming more prevalent due to global warming, deforestation, and global travel and trade [31]. Given these factors, it is expected that novel viruses will continue to be identified, potentially leading to their dissemination to non-endemic regions. This emphasizes the potential risks posed by vector-borne Simbu serogroup viruses to spread globally. Our study underscores the significance of employing genus-specific PCRs for orthobunyaviruses in surveillance, as these methods can detect lesser-known viruses that are not endemic to a given area. Additionally, we emphasize the importance of a comprehensive analysis of all three genome segments when characterizing orthobunyaviruses.

We present the foundation for further research into the prevalence and epidemiology of SHAV in SA. our study, there was no evidence of SHAV detection in animal abortions in the region. Our arbovirus surveillance efforts detected SHAV in *Culicoides* midges in Gauteng and Limpopo provinces between 2012 and 2017 [25], marking the first detection of SHAV in SA. This identification of SHAV in an aborted goat fetus from the Western Cape province suggests that the virus circulates wider and highlights the need to consider SHAV as a potential contributor to outbreaks of animal abortion in the region [15,20,32].

It is important to note that this study’s scope is specific to a single case, representing one of fifty instances of abortion within two weeks. While it suggests a potential association of SHAV with animal abortions during that time, further investigation is warranted, particularly in the Western Cape, to confirm definitive associations and evaluate its wider prevalence. Vector and serological studies could offer insights into its distribution and mode of transmission.

SHAV has not been detected for almost 40 years after its initial detection in Nigeria, but the current detection in SA between 2012 to 2017 and 2021 and in Japan in 2015 [23,33] suggests it may have the potential to emerge wider. With documented instances of causing severe signs such as torticollis, spinal curvature, abortions, and stillbirths in animals [33], further investigations are required to determine the potential zoonotic implications, similar to what was observed for SHUV in humans, horses and wildlife animals from natural habitats and managed environments [15,20,32]. Additionally, the absence of a vaccine candidate for SHAV in animals highlights the need for further research into the development of preventative or treatment options to mitigate the impact of an outbreak, emphasizing the importance of continued surveillance for emerging vector-borne viruses.

## 5. Conclusions

This study contributes to understanding the distribution, diversity, and potential implications of Simbu serogroup viruses in SA. Through a syndromic surveillance program targeting arboviruses in animals, we identified and characterized a SHAV strain in the brain of an aborted goat fetus from the Western Cape province in SA. The molecular analysis of this SHAV strain reveals its close relatedness to strains previously identified in Japan and *Culicoides* midges in the Gauteng and Limpopo provinces in SA in recent years. The molecular insights from this study will contribute to our broader understanding of these viruses and their potential to impact animal and human health. Continued collaborative efforts across disciplines, including virology, entomology, and epidemiology, are necessary to address these evolving challenges effectively.

## Figures and Tables

**Table 1:** Details of the oligonucleotides used for the amplification of partial S, M and L segments.

**Simbu Serogroup TaqMan Real-Time RT-PCR Assay**						
**Segment**	**Oligonucleotide Name**		**Target Size**	**Oligonucleotide Sequence (5′–3′)**	**Position**	**Reference**
Small	Ortho(SimbuF152)		152 base pairs (bp)	TAGAGTCTTCTTCCTCAAYCAGAAGAAGGCC	152–304	[20]
	Ortho(SimbuR304)			GTYAMGGCAMTGTCTGGCACAGGATTTG		
	Ortho(Simbu-probe252)			TGGTTAATAACCATTTTCC		
**Simbu serogroup conventional nested PCR assay**						
**Segment**	**Round**	**Oligonucleotide name**	**Target size**	**Oligonucleotide sequence (5′–3′)**	**Position**	**Reference**
Small	First	Ortho(SimbuF141)	576 bp	CGRTRYYGYTAGAGTCTTCTTCC	141–718	This study
		Ortho(SimbuR718)		CGAATTGGGCAAGGAAAGT		
	Nested	Ortho(SimbuFN403)	291 bp	CCNCTTGCTGARGTNAARG	403–695	
		Ortho(SimbuRN695)		GCAGCWGGWGAGAATCCWGA		
**Simbu serogroup conventional hemi-nested PCR assay**						
**Segment**	**Round**	**Oligonucleotide name**	**Target size**	**Oligonucleotide sequence (5′–3′)**	**Position**	**Reference**
Medium	First	SimbuM2671+	807 bp	CATCMAGATATWGAAWMHTWYATAKCAG	2671–3476	This study
		SimbuM3476-		GCATGRCAWAYATAATCAAATYKNGG		
	Nested	SimbuM2671+	421 bp	CATCMAGATATWGAAWMHTWYATAKCAG	2671–3110	
		SimbuM3110-		CCCCATTCTTCRCARCCC		
**Pan-orthobunyavirus hemi-nested PCR assay**						
**Segment**	**Round**	**Oligonucleotide name**	**Target size**	**Oligonucleotide sequence (5′–3′)**	**Position**	**Reference**
Large	First	Peri-F1	513 bp	CAAARAACAGCAAAAGAYAGRGARA	2907–3427	[27]
		Peri-R1		TTCAAATTCCCYTGIARCCARTT		
	Nested	Peri-F2	402 bp	ATGATTAGYAGRCCDGGHGA	3018–3427	
		Peri-R1		TTCAAATTCCCYTGIARCCARTT		

**Table 2:** Demographics of Case 3.

Laboratory Number	Animal	Clinical Signs	Date of Receipt	Type of Specimen	Sampling Location	Defined Virus
ZRU093/21	Goat	Aborted	5 May 2021	Brain, lung	Velddrif, Western Cape	Shamonda

## Data Availability

Additional data is available from the corresponding author.

## Supplementary Materials

The following supporting information can be downloaded at: https://www.mdpi.com/article/10.3390/pathogens12091100/s1, Table S1: Details on the procedures for the PCR assays used for the amplification of partial S, M and L segments; Table S2: Small and large segment primers used for amplicon sequencing of Shamonda virus in specimens from an aborted goat fetus (ZRU093/21); Table S3: Nucleotide and amino acid pairwise distances comparing the partial S segment fragment of Shamonda virus (ZRU093/21) and selected reference strains; Table S4: Nucleotide and amino acid pairwise distances comparing the partial M segment fragment of Shamonda virus (ZRU093/21) and selected reference strains; Table S5: Nucleotide and amino acid pairwise distances comparing the partial L segment fragment of Shamonda virus (ZRU093/21) and selected reference strains; virus (ZRU093/21) and selected reference strains. Figure S1: The initial detection of Shamonda virus (SHAV) in the brain and lung of the aborted goat fetus (ZRU093/21) using the Simbu serogroup TaqMan real-time RT-PCR assay. Curves 1, 2 and 3 represent the Shuni virus control, the brain specimen of ZRU093/21 and the lung specimen of ZRU093/21, respectively. The cycle threshold values for the brain and lung specimens were 29.68 and 36.25, respectively.
