# Supplementary material for: Identification and Molecular Characterization of Shamonda Virus in an Aborted Goat Fetus in South Africa
Source: Pathogens. 2023 Aug 28;12(9):1100. doi: 10.3390/pathogens12091100 (PMC10536486; doi:10.3390/pathogens12091100)
Supplement: Supplementary file 1 [file pathogens-12-01100-s001.zip › pathogens-2510072-supplementary.pdf]

Supplementary Table S1. Details on the procedures for the PCR assays used for the amplification of partial S, M and L segments.

Segment	PCR assay	Details on the procedure
Small	Simbu serogroup TaqMan real-time RT-PCR	The AgPath-ID™ One-Step real-time RT-PCR kit (Thermo Fisher Scientific, Massachusetts, USA) was used with the PCR master mix containing 0.45 µL of each primer (20 µM) and 0.25 µL of the probe (12 µM), 12.5 µL of 2X AgPath reaction mixture, 1 µL of 25X AgPath enzyme mixture and 10 µL of RNA, with a final volume of 25 µL. The PCR reactions were incubated at 50°C for 30 min, 94°C for 2 min followed by 40 cycles of 94°C for 15 seconds (s) and 58°C for 1 min.
	Simbu serogroup conventional nested RT-PCR	This PCR assay was performed by using the Superscript III One-Step RT-PCR System with Platinum Taq DNA Polymerase (Invitrogen, Thermo Fisher Scientific, Massachusetts, USA) for the first PCR and the Platinum™ Taq DNA Polymerase, DNA-free kit (Invitrogen, Thermo Fisher Scientific, Massachusetts, USA) for the nested PCR. The PCR master mix for the first PCR was prepared by adding 12.5 µL of 2X Reaction mix, 0.5 µL of the primers (20 µM), 0.5 µL of nuclease-free water, 1 µL of the SuperScript III/Platinum Taq mix, and 10 µL of RNA to a final volume of 25 µL. The PCR reactions were incubated at 50°C for 30 min, 94°C for 7 min, 35 cycles of 94°C for 30 s, 54°C for 1 min and 68°C for 1 min, a final extension of 68°C for 5 min and cooling down at 4°C. Volumes of 2.5 µL of 10X PCR Buffer, 0.75 µL of $MgCl_2$, 0.5 µL of dNTPs, 1 µL of both primers (20 µM), 17.0 µL of nuclease-free water, 0.25 µL of the Platinum Taq enzyme and 2 µL of the first-round product were mixed and had a total volume of 25 µL. The PCR reaction conditions for the nested PCR were as follows: 95°C for 3 min, followed by ten touch-down cycles at 98°C for 15 s, 51°C for 20 s and 72°C for 40 s, 35 cycles at 98°C for 15 s, 45°C for 20 s and 72°C for 40 s, 72°C for 10 min and cooling at 4°C.
Medium	Simbu serogroup conventional hemi-nested RT-PCR	The Superscript III One-Step RT-PCR System with Platinum Taq DNA Polymerase was used for the first round PCR. Components added together were 12.5 µL of 2X Reaction mix, 0.5 µL of the primers (20 µM), 0.5 µL of nuclease-free water, 1 µL of the SuperScript III/Platinum Taq mix, and 10 µL of RNA to a final volume of 25 µL. PCR reaction conditions were as follows: 50°C for 30 min, 94°C for 2 min, 40 cycles of 94°C for 15 s, 45°C for 30 s, and 68°C for 50 s followed by a final extension of 68°C for 5 min and a cooling down step of 4°C. The Platinum™ Taq DNA Polymerase, DNA-free kit was used for the nested PCR with the 25 µL PCR master mix consisting of 2.5 µL of 10X PCR Buffer, 0.75 µL of $MgCl_2$, 0.5 µL of dNTPs, 1 µL of both primers (20 µM), 16.0 µL of nuclease-free water, 0.25 µL of the Platinum Taq enzyme and 3 µL of the first-round product. These PCR reactions were incubated at 94°C for 2 min, 40 cycles of 94°C for 30 s, 45°C for 30 s, and 72°C for 30 s, extending for an additional 5 min at 72°C and cooling down to 4°C.

Large	Pan-orthobunyavirus conventional hemi-nested PCR	The first round PCR was conducted by using the SuperScript III One-Step RT-PCR System with Platinum Taq DNA Polymerase and by adding 12.5 μL of 2X Reaction mix, 0.5 μL of the primers (10 μM), 0.5 μL of nuclease-free water, 1 μL of the SuperScript III/Platinum Taq mix, and 10 μL of RNA to a final volume of 25 μL. Complementary deoxyribonucleic acid (cDNA) was synthesized by incubating the PCR mixture at 50°C for 30 min which was followed by denaturation at 95°C for 3 min, ten touch down cycles at 95°C for 15 s, 55°C for 20 s and 72°C for 40 s, followed by 35 cycles of 95°C for 15 s, 50°C for 20 s, and 72°C for 40 s, and a further extension of 72°C for 10 min ending with a 4°C cooling step. The hemi-nested PCR was performed by using the Platinum™ Taq DNA Polymerase, DNA-free kit and by mixing 2.5 μL of 10X PCR Buffer, 0.75 μL of $MgCl_2$, 0.5 μL of dNTPs, 1 μL of both primers (10 μM), 16 μL of nuclease-free water, 0.25 μL of the Platinum Taq enzyme and 3 μL of template (first-round PCR product). The PCR reaction conditions were similar to the first-round PCR excluding the cDNA synthesis step at 50°C for 30 min.

Supplementary Table S2. Small and large segment primers used for amplicon sequencing of Shamonda virus in specimens from an aborted goat fetus (ZRU093/21).

Small segment			
**Primer pool 1**	**Primer sequence**	**Primer pool 2**	**Primer sequence**
SimbuSF1	ATGTCAAGCCAATTCATTTTYGAAG	SimbuSF2	TDGGMTTYGCACCDGGNG
SimbuSR1	CTVTGCATHTCRATRACYARHGGRTARAAV	SimbuSR2	TTAGATDTTDATRCCRAATTGVKSMARG
**Large segment**			
**Primer pool 1**	**Primer sequence**	**Primer pool 2**	**Primer sequence**
SimbuLF1	ATGGAGACATACAARATHAAYATYTTTAG	SimbuLF2	TYYTRGARATATTYGGBCCRCTYAAC
SimbuLR1	TATATYARKGAYCGYARRTTRAARAACC	SimbuLR2	GYTKRCCCCATATGAARTGRAYAG
SimbuLF3	GTRAGTRYDGARMGAAAYCTKACTCARG	SimbuLF4	YCTTGGYATAGGRGGYCAYG
SimbuLR3	TTTRTTRAAATCYARDATAGTAGGTTTRTC	SimbuLR4	GARCCWGWYTKRAATGTYGCATG
SimbuLF5	TRGCTGTRTTRCATTCTRSYYCYC	SimbuLF6	TAYATGGCATATARTCARAGRGAYAAAG
SimbuLR5	TTTGRGTTATYTCATAATCWGTYARRC	SimbuLR6	TTTCYTTYTCAAARTYBCCYACYTTRATRC
SimbuLF7	AGRTCRATATGCACTATTTCTACATTY	SimbuLF8	RTATYTAGTRGARAGRATATCYAAAGAACG
SimbuLR7	RTCGCCTGGYTCRCTRATC	SimbuLR8	TARTTRAGATTCCCYTGCARCC
SimbuLF9	ATGARATGACYAAYGGYTTRTCRC	SimbuLF10	TATGARGATGTTGCCAGYAG
SimbuLR9	AKGCAAKNGADGGHGGTGC	SimbuLR10	DGCAGTRGTRAAYTTYCTYGGTG
SimbuLF11	TCTGATGAYGGRATGGGYG	SimbuLF12	AAGACYTTTRTTGARACATAYCAGC
SimbuLR11	ACAGAARCTRWAWATYGTYTTTATRTCYTC	SimbuLR12	GTTCYGTYGAYTTHACATAYTCATARC
SimbuLF13	GATYTGAARTTTGARGTGCARG	SimbuLF14	RCATAYACATAYAARGAYGTYCAAGTRTC
SimbuLR13	TTTCTGCATCAAAYCTRTCAAYRTC	SimbuLR14	CBRGWBGCHWSWATYYWWGRRKTRTG
SimbuLF15	KRAASMMRTDRYYGAYYTRAWYYCHWARTK	SimbuLF16	WCWDRRTWTHACRTKRYTHGAYTTRWKCYG
SimbuLR15	YAVRAWTTCTAGRAATSYATCWTSATCYAY	SimbuLR16	YYSTKKCWYKYRRTRYVTYWWMRRRCWTRM
SimbuLF17	WWYTSWNGAYRWYMSMAWNYMRHWRMMWDY	SimbuLF18	CATAGGAGGCCATGTGC
SimbuLR17	AAATCTAGGATAGTAGGTTTATCAG	SimbuLR18	CTTAGAACCTGTCTTAAATGTTG

The M segment primers designed for next-generation amplicon sequencing did not result in any viral sequences and were, therefore, not included in the supplementary material.

Supplementary Table S3. Nucleotide and amino acid pairwise distances comparing the partial S segment fragment of Shamonda virus (ZRU093/21) and selected reference strains.

Nucleotide<br>Amino acid	AINOV	PEAV	SANV	SHUV	AKAV	TINV	SABV	SBV	SHAV	ZRU093/21	SATV	DOUV	SIMV	OROV
**AINOV**		0.09	0.08	0.05	0.23	0.23	0.22	0.23	0.23	0.24	0.22	0.22	0.21	0.32
**PEAV**	0.20		0.08	0.08	0.23	0.24	0.24	0.23	0.23	0.23	0.23	0.22	0.22	0.32
**SANV**	0.20	0.20		0.09	0.23	0.23	0.23	0.22	0.23	0.23	0.23	0.22	0.21	0.31
**SHUV**	0.12	0.18	0.23		0.23	0.23	0.23	0.23	0.23	0.23	0.22	0.22	0.21	0.32
**AKAV**	0.49	0.45	0.49	0.49		0.05	0.19	0.23	0.23	0.23	0.21	0.22	0.20	0.31
**TINV**	0.48	0.47	0.47	0.47	0.13		0.17	0.23	0.24	0.23	0.22	0.23	0.21	0.31
**SABV**	0.47	0.49	0.49	0.47	0.40	0.35		0.21	0.21	0.21	0.21	0.20	0.22	0.32
**SBV**	0.48	0.48	0.44	0.48	0.43	0.44	0.42		0.02	0.04	0.07	0.08	0.22	0.31
**SHAV**	0.49	0.47	0.47	0.48	0.44	0.47	0.40	0.07		0.03	0.07	0.07	0.21	0.31
**ZRU093/21**	0.50	0.47	0.46	0.49	0.43	0.45	0.40	0.12	0.08		0.07	0.08	0.22	0.31
**SATV**	0.45	0.47	0.46	0.47	0.41	0.41	0.41	0.19	0.19	0.18		0.07	0.21	0.32
**DOUV**	0.42	0.44	0.44	0.43	0.42	0.43	0.37	0.21	0.19	0.21	0.17		0.19	0.31
**SIMV**	0.41	0.43	0.42	0.41	0.44	0.45	0.45	0.41	0.40	0.42	0.40	0.37		0.31
**OROV**	0.61	0.60	0.57	0.59	0.54	0.54	0.60	0.51	0.52	0.52	0.56	0.53	0.56	

AINOV: Aino virus (MH484278.1); PEAV: Peaton virus (MH484320.1); SANV: Sango virus (MZ285924.1); SHUV: Shuni virus (MW729741.1); AKAV: Akabane virus (LC552050.1); TINV: Tinaroo virus (MH484341.1); SABV: Sabo virus (NC_043554.1); SBV: Schmallenberg virus (NC_043582.1); SHAV: Shamonda virus (LC741389.1); ZRU093/21 (OR249959.1); SATV: Sathuperi virus (NC_018462.1); DOUV: Douglas virus (HE795092.1); SIMV: Simbu virus (NC_018477.1); OROV: Oropouche virus (KP026181.1).

Supplementary Table S4. Nucleotide and amino acid pairwise distances comparing the partial M segment fragment of Shamonda virus (ZRU093/21) and selected reference strains.

Nucleotide<br>Amino acid	AINOV	SHUV	SBV	DOUV	SATV	SIMV	AKAV	TINV	SABV	SHAV	ZRU093/21	PEAV	SANV	OROV
**AINOV**		0.28	0.40	0.37	0.39	0.36	0.48	0.48	0.45	0.50	0.50	0.49	0.46	0.46
**SHUV**	0.59		0.39	0.37	0.39	0.36	0.47	0.48	0.44	0.46	0.45	0.43	0.42	0.41
**SBV**	0.69	0.67		0.18	0.17	0.39	0.47	0.48	0.44	0.46	0.47	0.44	0.46	0.45
**DOUV**	0.68	0.63	0.45		0.15	0.39	0.47	0.48	0.46	0.46	0.46	0.46	0.44	0.44
**SATV**	0.72	0.67	0.38	0.39		0.39	0.45	0.47	0.44	0.44	0.44	0.43	0.43	0.44
**SIMV**	0.70	0.68	0.72	0.77	0.77		0.48	0.42	0.42	0.45	0.44	0.43	0.44	0.41
**AKAV**	0.80	0.74	0.68	0.69	0.65	0.76		0.35	0.34	0.39	0.40	0.39	0.42	0.45
**TINV**	0.76	0.77	0.73	0.81	0.74	0.75	0.61		0.32	0.38	0.38	0.41	0.41	0.46
**SABV**	0.74	0.69	0.73	0.76	0.75	0.74	0.68	0.64		0.40	0.40	0.40	0.40	0.44
**SHAV**	0.81	0.74	0.72	0.72	0.70	0.77	0.69	0.66	0.70		0.02	0.39	0.38	0.47

**ZRU093/21**	0.78	0.72	0.72	0.72	0.70	0.75	0.70	0.66	0.70	0.06		0.40	0.38	0.46
**PEAV**	0.77	0.75	0.65	0.72	0.64	0.74	0.63	0.70	0.68	0.67	0.68		0.15	0.45
**SANV**	0.70	0.68	0.65	0.68	0.63	0.76	0.68	0.70	0.75	0.63	0.64	0.36		0.43
**OROV**	0.76	0.72	0.71	0.70	0.75	0.70	0.69	0.70	0.72	0.71	0.69	0.76	0.69	

AINOV: Aino virus (NC_018459.1); SHUV: Shuni virus (KU937312.1); SBV: Schmallenberg virus (NC_043584.1); DOUV: Douglas virus (HE795091.1); SATV: Sathuperi virus (LC741385.1); SIMV: Simbu virus (NC_018478.1); AKAV: Akabane virus (NC_009895.1); TINV: Tinaroo virus (MH484340.1); SABV: Sabo virus (NC_043552.1); SHAV: Shamonda virus (LC741388.1); ZRU093/21 (OR249960.1); PEAV: Peaton virus (HE795094.1); SANV: Sango virus (NC_043555.1); OROV: Oropouche virus (MF926353.1).

Supplementary Table S5. Nucleotide and amino acid pairwise distances comparing the partial L segment fragment of Shamonda virus (ZRU093/21) and selected reference strains.

**Nucleotide<br>Amino acid**	**AINOV**	**SHUV**	**PEAV**	**SANV**	**SIMV**	**AKAV**	**TINV**	**SABV**	**SBV**	**SHAV**	**ZRU093/21**	**DOUV**	**SATV**	**OROV**
**AINOV**		0.14	0.21	0.20	0.31	0.32	0.31	0.32	0.31	0.31	0.32	0.31	0.33	0.37
**SHUV**	0.44		0.22	0.21	0.32	0.32	0.31	0.32	0.33	0.33	0.33	0.33	0.33	0.36
**PEAV**	0.67	0.70		0.15	0.32	0.32	0.32	0.33	0.31	0.32	0.32	0.32	0.32	0.36
**SANV**	0.61	0.68	0.45		0.31	0.34	0.34	0.33	0.31	0.32	0.33	0.32	0.30	0.37
**SIMV**	0.87	0.94	0.89	0.93		0.32	0.32	0.31	0.32	0.32	0.32	0.32	0.32	0.37
**AKAV**	0.94	0.98	0.94	1.02	0.89		0.12	0.28	0.31	0.32	0.32	0.32	0.32	0.36
**TINV**	0.84	0.84	0.90	0.94	0.87	0.41		0.27	0.31	0.31	0.31	0.31	0.32	0.37
**SABV**	0.97	0.90	0.98	0.99	0.84	0.91	0.91		0.33	0.32	0.34	0.32	0.33	0.38
**SBV**	0.88	0.92	0.89	0.94	0.90	0.90	0.91	0.94		0.09	0.09	0.15	0.15	0.36
**SHAV**	0.82	0.93	0.89	0.95	0.87	0.87	0.82	0.88	0.29		0.03	0.13	0.14	0.36
**ZRU093/21**	0.87	0.93	0.89	0.99	0.87	0.87	0.85	0.94	0.29	0.09		0.15	0.14	0.35
**DOUV**	0.84	0.90	0.95	0.93	0.96	0.93	0.90	0.94	0.49	0.41	0.46		0.13	0.37
**SATV**	0.97	1.00	0.85	0.83	0.93	0.89	0.96	0.98	0.56	0.47	0.47	0.39		0.37
**OROV**	1.01	1.00	1.02	1.02	1.08	1.09	1.11	1.12	1.09	1.04	1.01	1.08	1.06	

AINOV: Aino virus (NC_018465.1); SHUV: Shuni virus (NC_043699.1); PEAV: Peaton virus (MH484318.1); SANV: Sango virus (MZ285922.1); SIMV: Simbu virus (NC_018476.1); AKAV: Akabane virus (KY284021.1); TINV: Tinaroo virus (MH484339.1); SABV: Sabo virus (NC_043553.1); SBV: Schmallenberg virus (NC_043583.1); SHAV: Shamonda virus (LC741387.1); ZRU093/21 (OR249961.1); DOUV: Douglas virus (HE795090.1); SATV: Sathuperi virus (LC741384.1); OROV: Oropouche virus (KP691603.1).

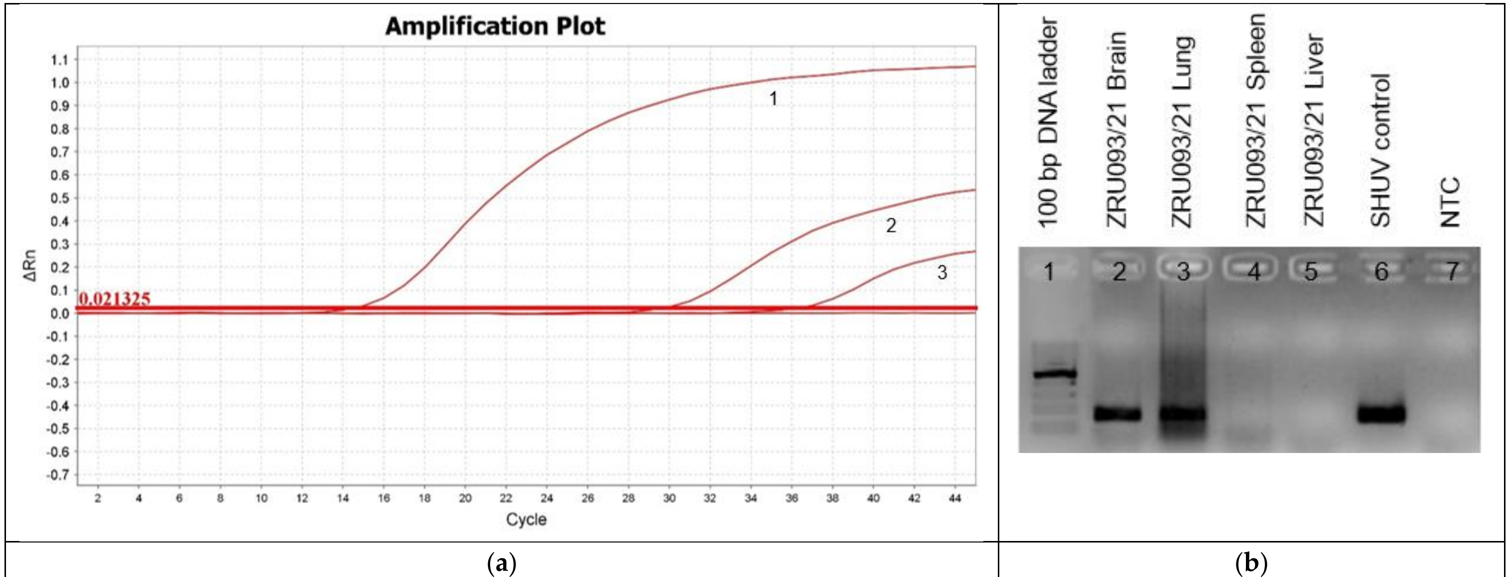

(**a**) (**b**)

Figure S1. The initial detection of Shamonda virus (SHAV) in the brain and lung of the aborted goat fetus (ZRU093/21) using the Simbu serogroup TaqMan real-time RT-PCR assay. Curves 1, 2 and 3 represent the Shuni virus control, the brain specimen of ZRU093/21 and the lung specimen of ZRU093/21, respectively. The cycle threshold values for the brain and lung specimens were 29.68 and 36.25, respectively.
